# Differences in novel *Schistosoma mansoni* Sm25 and Sm29 rapid diagnostic test performance based on sample matrix

**DOI:** 10.3389/fpara.2026.1891989

**Published:** 2026-08-07

**Authors:** Madelaine Usey Talbot, KathyJean Farnam, Jean Saunders, E. Brook Goodhew, Gabrielle Smith, Sylvia Ossai, Yong Wang, Sukwan Handali, Isaac O. Onkanga, Pauline N. Mwinzi, Maurice R. Odiere, Matthias Schwarz, Marco Biamonte, W. Evan Secor

**Affiliations:** 1 Laboratory Leadership Service, Division of Workforce Development, Public Health Infrastructure Center, Centers for Disease Control and Prevention, Atlanta, GA, United States; 2 Drugs and Diagnostics for Tropical Diseases, San Diego, CA, United States; 3 Division of Parasitic Diseases and Malaria, National Center for Emerging and Zoonotic Infectious Diseases, Centers for Disease Control and Prevention, Atlanta, GA, United States; 4 CDC Foundation, Atlanta, GA, United States; 5 Centre for Global Health Research, Kenya Medical Research Institute, Kisumu, Kenya

**Keywords:** rapid diagnostic test (RDT), sample matrix, *Schistosoma mansoni*, schistosomiasis, Sm25, Sm29, Target Product Profile (TPP), transmission interruption and surveillance

## Abstract

**Introduction:**

Schistosomiasis is a neglected tropical disease caused by parasitic worms of the genus *Schistosoma*. As schistosomiasis control programs approach elimination as a public health problem, there is an urgent need for sensitive diagnostic assays that can easily be deployed in the field at low cost. For schistosomiasis transmission interruption and surveillance use cases, the diagnostic Target Product Profile (TPP) proposed a two-step testing strategy involving an initial point-of-care screening test capable of detecting either current or former infections with sensitivity > 0.95, specificity > 0.80, and result stability ≥ 0.5 hours. To meet this need, Drugs and Diagnostics for Tropical Diseases designed two novel rapid diagnostic test (RDT) prototypes that detect antibodies against recombinant *Schistosoma mansoni* antigens: Sm25 and Sm29.

**Methods:**

We evaluated the performance of these RDT prototypes using serum, whole blood, and dried blood spots (DBS). RDT results were determined by two visual readers and a Lumos automated reader, which also provided a quantitative readout of test line intensity. Lumos results were used to quantify test line stability over time. Further, we compared RDT results to other serologic assays (ELISA and the multiplex bead assay) that detect antibodies against Sm25, Sm29 and two additional *S. mansoni* antigens.

**Results:**

While both Sm25 and Sm29 RDTs exhibited high specificity and stability that surpassed TPP criteria, their sensitivity fell short of TPP goals. Sensitivity was highest with serum, followed by DBS, then whole blood, which would be the most useful for point-of-care settings. In addition, we identified a gap in the ability of currently used recombinant antigens to detect all people infected with *S. mansoni*, regardless of test platform.

**Discussion:**

Our findings underscore that while these prototypes represent a promising start, there is still a case for novel antigen discovery and generation of RDTs with increased sensitivity to meet TPP criteria.

## 1 Introduction

Schistosomiasis is a neglected parasitic disease of humans caused by five species of blood flukes in the genus *Schistosoma*. Depending on the species, infection with *Schistosoma* spp. can result in either intestinal or urogenital disease, both of which are responsible for disability or even death in severe cases. While the distribution of schistosome species varies geographically, over 250 million people required preventive treatment for schistosomiasis in 2024 (WHO, 2025). The species that is the primary cause of intestinal schistosomiasis worldwide, *Schistosoma mansoni*, is found throughout Africa, the Middle East, South America, and some areas of the Caribbean. Control programs for schistosomiasis are based on mass drug administration (MDA) with praziquantel, improved access to sanitation and clean water, proper hygiene education, and control of intermediate host snails (WHO, 2023).

For over 50 years, decisions about *S. mansoni* control programs have primarily been made using prevalence data based on the Kato-Katz technique, which involves microscopic examination of human stool to identify *S. mansoni* eggs (Katz et al., 1972). While useful in areas of high prevalence, Kato-Katz lacks sensitivity when infections are light, which is of particular concern in low transmission areas. While collecting multiple stools can increase sensitivity by overcoming day-to-day variability in egg counts and stool consistency, this creates logistical issues and increases costs (Teesdale et al., 1985, de Vlas and Gryseels, 1992, Booth et al., 2003, Barenbold et al., 2017). This lack of sensitive, easy to use, low-cost tests has hindered progress towards schistosomiasis transmission interruption. To direct the development of future tests, the Diagnostic Technical Advisory Group for Neglected Tropical Diseases (DTAG-NTD) was established in 2019 to create target product profiles (TPPs) for schistosomiasis and other NTDs. The schistosomiasis TPPs provide sets of characteristics to guide the development of tests for (1) monitoring and evaluation of ongoing schistosomiasis control programs and for (2) determining whether transmission has been interrupted and post-MDA surveillance can be conducted (WHO, 2021).

Diagnostic tests that meet the transmission interruption and surveillance TPP need the capability to detect a 3% infection prevalence, which requires a two-step testing strategy to achieve the necessary specificity (WHO, 2021). This strategy involves an initial, low-cost screening test with an ideal sensitivity above 0.95 and specificity above 0.80. The screening test could detect both former and current infections, i.e. an antibody detection test. For any individuals who test positive by the initial test, a confirmatory test for active infection with a sensitivity above 0.93 and a specificity above 0.98 would then be used (WHO, 2021). In this case, only individuals with a positive confirmatory test result would be considered actively infected. Lastly, at minimum, the initial test should be able to be performed at the point-of-care (POC) and results should be stable for at least 0.5 hours (WHO, 2021).

**Figure 1:**
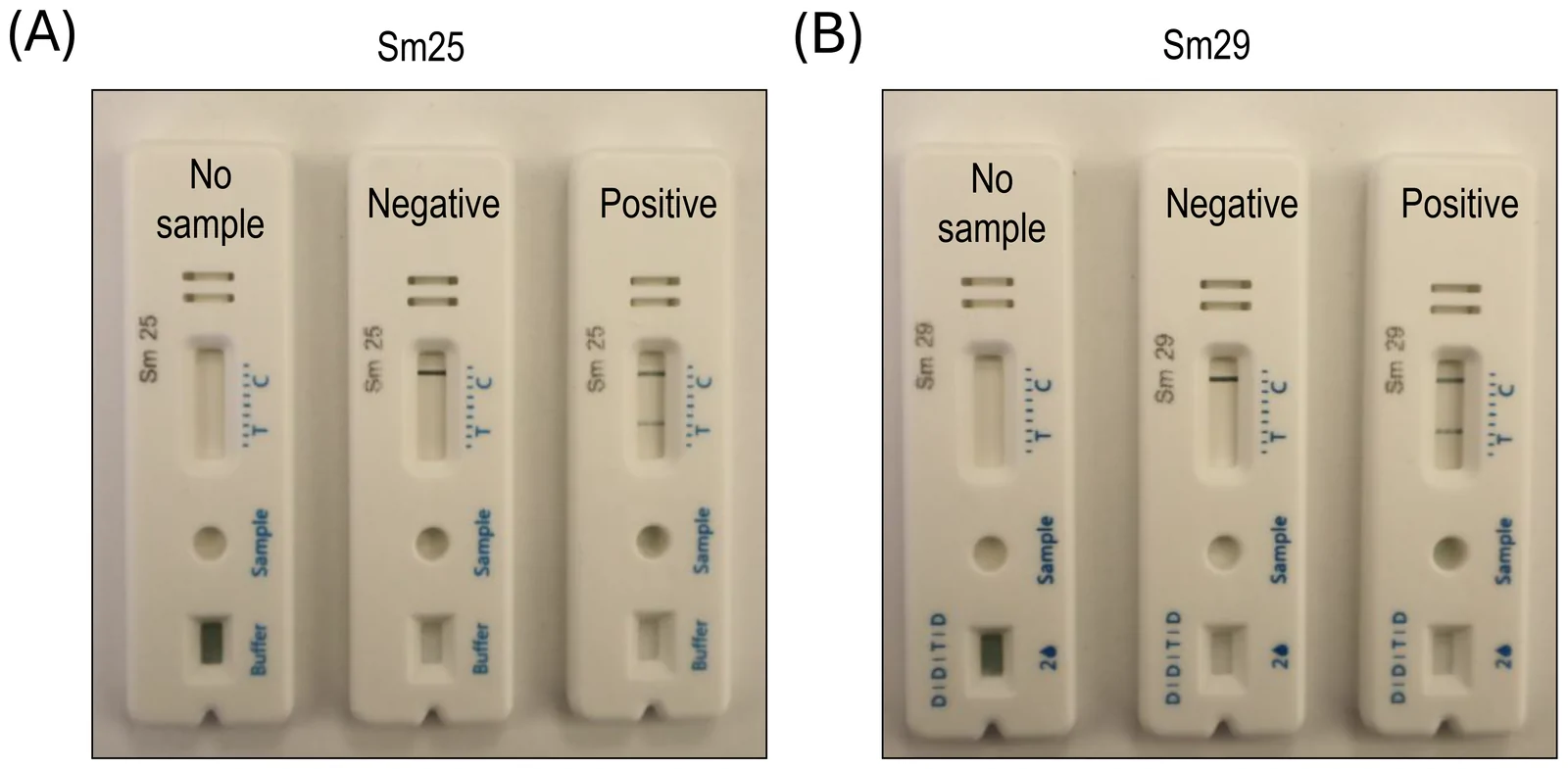
Key aspects of the DDTD Sm25 and Sm29 RDT cassette prototypes. Examples of both Sm25 **(A)** and Sm29 **(B)** RDTs are shown. From left to right: RDT before sample addition, negative RDT, positive RDT.

To meet the needs of an initial screening test for transmission interruption and surveillance (WHO, 2021), Drugs and Diagnostics for Tropical Diseases (DDTD) generated novel rapid diagnostic test (RDT) prototypes based on two recombinant proteins. These RDTs detect IgG1 antibodies against Sm25 and Sm29, two *S. mansoni* adult worm tegumental proteins, from a blood sample (**Figures 1A,B**) (Smithers et al., 1989, Ali et al., 1991, Cardoso et al., 2006). These two antigens have been used in other serological assays to detect *S. mansoni* infection (Tsang et al., 1984, Krautz-Peterson et al., 2017, Won et al., 2017, Carcelen et al., 2025).

To evaluate the sensitivity and specificity of Sm25 and Sm29 RDT prototypes, staff at the Centers for Disease Control and Prevention (CDC) tested a matched sample set consisting of three different matrices: serum, contrived whole blood, and contrived dried blood spots (DBS). Using these samples, we determined whether these prototype RDTs met TPP criteria for an initial screening test for use when schistosomiasis control programs approach interruption of transmission milestones or further surveillance is needed.

## 2 Materials and methods

### 2.1 Ethics

Use of de-identified serum specimens from persons infected with schistosomes or other parasitic diseases underwent human subjects review at CDC (Project ID: 0900f3eb81b9a95f, Accession #: CGH-LRDU-7/20/20-9a95f). The study was conducted consistent with applicable federal law and CDC policy (45 C.F.R. part 46.102(l)(2), 21 C.F.R. part 56; 42 U.S.C. Sect. 241(d); 5 U.S.C. Sect. 552a; 44 U.S.C. Sect. 3501 et seq.).

### 2.2 Preparation of specimens

Sera used in this work were derived from several previously conducted studies and activities. The sample set consisted of 50 *S. mansoni* egg-positive sera from schoolchildren in Kenya (Sircar et al., 2018), 50 *Schistosoma haematobium* egg-positive sera (Mansour et al., 1981), and 50 normal human sera from a non-endemic area (NHS) (Hancock and Tsang, 1986). The cross-reactor panel consisted of 39 total sera, including 8 sera from individuals infected with *Trypanosoma cruzi* for *Leishmania donovani*, and 12 sera from blood banks in Haiti identified as *Wuchereria b* (Rivera et al., 2022), 19 sera from individuals positive via clinical blood culture *ancrofti* positives. Positives for *S. mansoni* had *S. mansoni* eggs identified via Kato-Katz in at least one stool from specimens collected over three consecutive days and were negative for malaria via blood smear microscopy and for infection with *Trichuris trichiura*, *Ascaris lumbricoides*, and hookworm (if data were available) via Kato-Katz (Sircar et al., 2018). Average *S. mansoni* eggs per gram of stool (EPG) ranged from 8 to 884. Of these specimens, 30% (n=15) of *S. mansoni* positives were considered light infections (1–99 EPG), 44% (n=22) were considered moderate intensity infections (100–399 EPG), and 26% (n=13) were considered heavy intensity infections (>400 EPG). For all sensitivity and specificity analyses, the 50 *S. mansoni* egg-positives were considered positive while the 139 *S. haematobium*, parasite cross-reactor, and NHS samples were all considered negative.

The identities of all sera were blinded from the individuals conducting the study. To generate contrived whole blood and contrived DBS for each sample, whole blood from donors who have never been exposed to schistosomes was centrifuged to separate red blood cells (RBCs) from serum. The serum was removed, then RBCs were washed three times with equivalent volumes of phosphate-buffered saline (PBS) to ensure no serum remained. Following the final wash, PBS was removed, and equivalent amounts of each blinded serum sample were added to separate tubes with the RBCs to create contrived whole blood samples. After mixing, 10 µl of every contrived whole blood sample was pipetted onto 6 mm extensions of filter paper wheels (TropBio, cat. no. 05-002-12) and allowed to dry overnight to create contrived DBS. Once dry, DBS were packaged individually in sealed plastic bags and stored at -20 °C with desiccant. Prior to use, DBS were eluted for 1 hour at room temperature (RT) shaking at 700 rpm in 100 µl of DDTD’s proprietary RDT chase buffer.

### 2.3 Sm25 and Sm29 RDTs

Prototype RDT cassettes from DDTD (**Figure 1**) were placed on a flat surface prior to addition of sample (either 2.5 µl serum, 5 µl contrived whole blood, or 10 µl contrived eluted DBS) to the sample port. Next, 50 µl of DDTD chase buffer was added to the buffer port. After a 10-minute incubation, an additional 50 µl of chase buffer was added and allowed to incubate for an additional 20 minutes. Cassettes were immediately visually scored as positive (1), borderline positive (0.5), or negative (0) by two independent readers. Only cassettes with a visible control line were evaluated. Because of subjectivity surrounding borderline result calling, cassettes with a borderline (0.5) result by one operator and a positive (1) or negative (0) by the other operator were not considered discrepant. If a cassette had a positive (1) result by one operator and a negative (0) result by the other operator, results were considered discrepant and a third operator served as the tiebreaker. Positivity using visual results was calculated for two different categories. A cassette was considered ‘Visual – Positive Only’ if at least one reader scored the sample as positive. Cassettes in the ‘Visual – Borderline Included’ category included all cassettes in the 'Visual – Positive Only' category as well as those that at least one reader scored as borderline.

Cassettes were also evaluated using the Lumos reader (Lumos Diagnostics, cat. no. Lumos-V3-03). Any cassette with a test line peak greater than 0.01 relative units (RU) was considered positive. To set the positivity threshold, 20 negative samples from a non-endemic area and 10 microscopy-confirmed positive samples were tested on the RDTs and visually scored in a binary fashion (negative or positive) by 5 individual users. The test line intensities were also quantified using the Lumos benchtop reader. For the reader settings used for this testing, Test Line (TL) intensities <0.01 relative units had 100% agreement across all 5 users being visually scored as negative. TL intensities that measured >0.01 RU on the Lumos reader had at least 1/5 user visually score the result as positive.

Lumos values, in RU, were compared across paired serum, whole blood, and DBS sample matrices. Because values were not normally distributed for Sm25 and Sm29, paired matrix differences were evaluated using the Friedman test followed by paired Wilcoxon signed-rank tests with Bonferroni adjustment. The Bonferroni adjustment was conducted to limit the possibility that any significant p-values observed were a chance result of having performed multiple comparisons. For stability testing, RDT cassettes were stored at RT in sealed, clear plastic bags for approximately 60 days before they were evaluated again using the Lumos reader.

### 2.4 Enzyme-linked immunosorbent assays (ELISAs)

Immulon 2HB plates (Thermo Scientific, cat. no. 3455) were separately coated with the following *S. mansoni* recombinant antigens (custom-produced by Genscript) as previously described (Gwyn et al., 2017): Sm25, Sm29, and TSP2. Briefly, each antigen was diluted to 1 µg/mL in Sensitization Buffer (50 mM Tris-HCl, pH 8.0 + 2 mM ethylenediaminetetraacetic acid [EDTA] + 0.3 M KCl). Diluted antigens were added to each well of Immunlon 2HB plates then incubated overnight at 4 °C. The next day, plates were washed four times with PBST (1x PBS + 0.3% Tween-20) and blotted dry. StabilCoat (Surmodics IVD inc., cat. no. SC01-2000) was added to each well to block non-specific binding. Plates were incubated at RT for 30 minutes shaking at 600 rpm. Liquid in the wells was decanted and plates were dried at 30 °C for 4 hours at 0.73 **–** 0.90 atmospheres of pressure. Plates were stored in aluminum pouches with 1g silica desiccant at 4 °C until use.

To run the ELISAs, sera were diluted 1:100 in PBSTM (PBST + 5% [w/v] dry skim milk) then each sample was added in duplicate to the pre-coated plate. A standard curve (0.625–20 ng/ml anti-Sm25 monoclonal antibodies [Medical and Biological Laboratories, Tokyo, Japan, custom-made], 0.325–20ng/ml anti-Sm29 monoclonal antibodies [Medical and Biological Laboratories, Tokyo, Japan, custom-made], or 1–30 arbitrary units of pooled positive sera for TSP2), positive controls, negative controls, and blanks were added to each plate in duplicate. Samples were incubated for 30 minutes shaking 600 rpm at RT then washed with PBST using a Biotek 405 TS automated plate washer (Agilent, cat. no. 405TSR-SN). Horseradish peroxidase (HRP)-conjugated mouse anti-human IgG1 (Southern Biotech, cat. no. 9052-05) was diluted 1:1000 in PBSTM then added to each well and incubated 30 minutes shaking at RT. Wells were washed before the addition of tetramethylbenzidine (TMB) SureBlue™ (Seracare, cat. no. 5120-0076). After shaking for 10 minutes at RT, TMB BlueSTOP (Seracare, cat. no. 5150-0022) was added to each well. Plates were read on a Biotek Synergy HTX multi-mode plate reader (Agilent, cat. no. S1LFA) at an absorbance of 650 nm and analyzed using Gen5 software. Positivity cutoffs were set at 0.89 units/µl for Sm25, 0.52 units/µl for Sm29, and 2.9 units/µl for TSP2. Cutoffs were established using a receiver-operating characteristic curve based on a panel of previously identified positive and negative sera. Any samples within 10% +/- of the positivity cutoff or with discordant technical replicates were re-tested until concordance was reached.

### 2.5 Antigen coupling to Luminex microspheres

Antigens (Sm25, Sm29, TSP2, and *S. mansoni* soluble egg antigen [SEA; in-house]) were coupled to MagPlex**^®^** Microsphere beads (Luminex, cat. no. MC100xx-01 or -04) with slight modifications to previously described methods (Goodhew et al., 2012). Briefly, beads were washed using a 0.1 M NaH_2_PO_4_, pH 6.2 (NaP) buffer. Carboxyl groups on the beads were modified to ester groups using a solution of 5 mg/ml 1-ethyl-3-(3-dimethylaminopropyl) carbodiimide (Calbiochem, cat. no. 341006) and 5 mg/ml N-hydroxysulfosuccinimide (Pierce, cat. no. 24510) in NaP. The ester groups on 1.25 x 10^7^ beads were then allowed to react with primary amines on the antigens using the following amounts and in either 1x PBS pH 7.2 or 50 mM 2-(N-morpholinoethanesulfonic acid (MES) pH 5, 0.85% NaCl as indicated: Sm25 (12 µg, PBS), Sm29 (30 µg, MES), TSP2 (15 µg, MES), *S. mansoni* SEA (120 µg, PBS). Following this step, beads were washed in 1x PBS then blocked in a 1x PBS solution containing 1% bovine serum albumin (BSA). Beads were counted then stored at 4 °C in a solution of 1x PBS, 1% BSA, 0.05% Tween-20, 0.02% NaN_3_, 200 µg/ml Pefabloc SC (Millipore Sigma, cat. no. 76307-100MG), 1x Pierce Protease Inhibitor Tablet with EDTA (Pierce, cat. no. A32963).

### 2.6 Multiplex bead assay (MBA)

To prepare samples for MBA, sera were diluted at 1:400 in Buffer B (1x PBS, 0.5% casein, 0.5% polyvinyl alcohol, 0.8% polyvinylpyrrolidone, 0.3% Tween-20, 0.02% NaN_3_, and 3 µg/mL *Escherichia coli* extract) and allowed to incubate overnight at 4 °C. Diluted specimens were tested by MBA with minor modifications to the methods as previously described (Goodhew et al., 2012). Briefly, 1,000 beads/antigen in Buffer A (1x PBS, 0.5% BSA, 0.05% Tween 20, 0.02% NaN_3_) were added to each well of a 96-well filter bottom plate (Millipore, cat. no. MABVN1250). The beads were washed with PBST using vacuum filtration (Millipore, cat. no. MSVMHTS00). Each diluted specimen was incubated in duplicate with the beads for 1.5 hours at RT shaking at 700 rpm. After washing, beads were incubated with 50 ng mouse anti-human IgG (Southern Biotech, cat. no. 9042-08) and 40 ng mouse anti-human IgG4 (Southern Biotech, cat. no. 9200-08) in Buffer A shaking at RT for 45 minutes. Beads were washed then incubated with 250 ng streptavidin, R-phycoerythrin conjugate (SAPE) (Invitrogen, cat. no. S-866) in Buffer A for 30 minutes shaking at RT. After washing, beads were incubated overnight at 4 °C in 1x PBS. The next day, beads were read on the MAGPIX (Millipore, cat. no. 40-072). The average median fluorescence intensity (MFI) with background (Buffer B alone) subtracted (net MFI) was determined for each antigen for each specimen. A receiver-operating characteristic curve based on a panel of previously identified positive and negative sera was used to determine the net MFI positivity cutoff value for each antigen: SEA (181.5), TSP2 (28.5), Sm25 (20.5), and Sm29 (20).

## 3 Results

### 3.1 Diagnostic sensitivity and specificity of the Sm25 and Sm29 RDTs by Lumos reader

**Figure 2:**
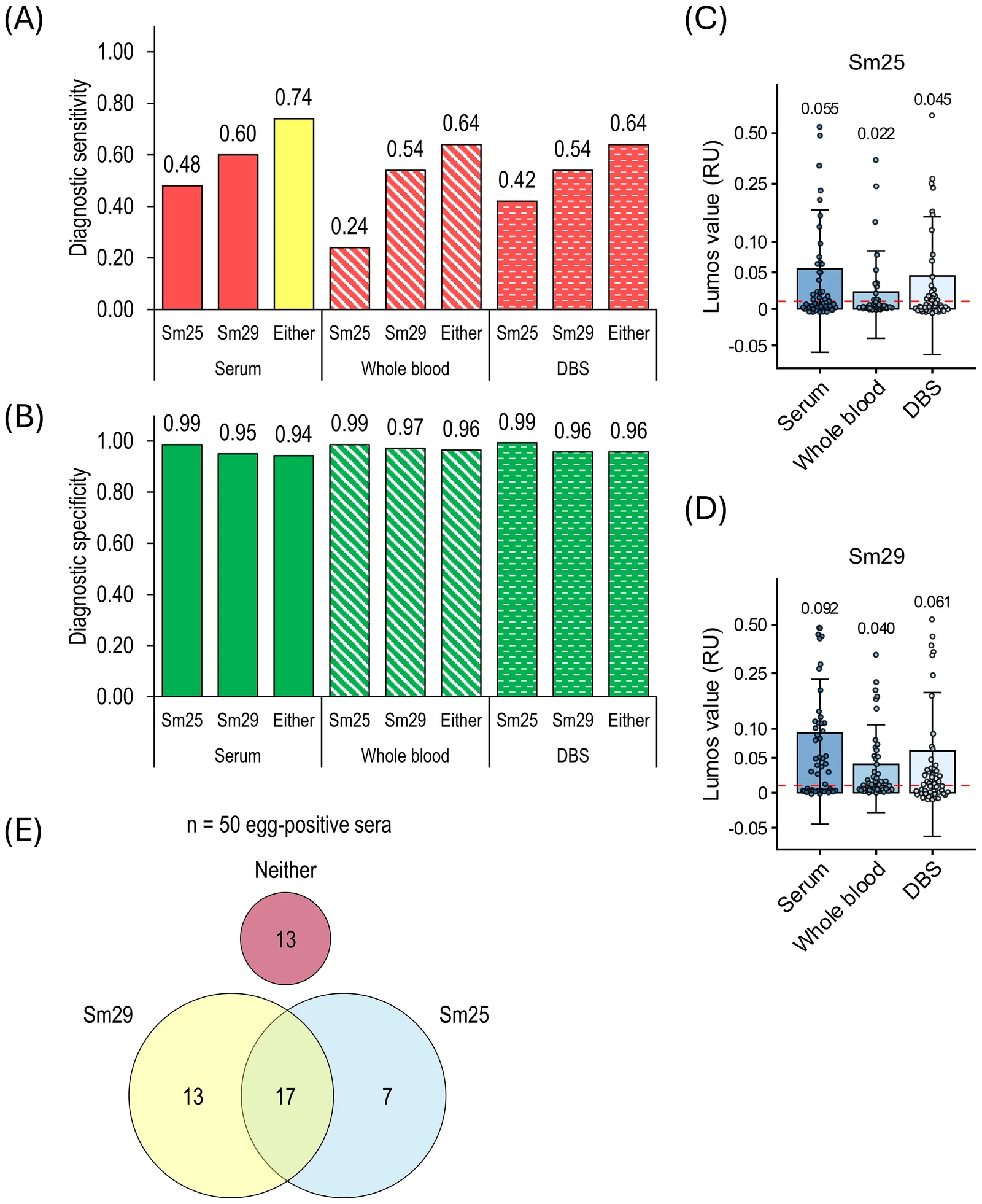
Diagnostic characteristics of the Sm25 and Sm29 RDTs as determined by the Lumos reader. Diagnostic sensitivity **(A)** and specificity **(B)** were calculated for all three sample matrices (serum, whole blood, and DBS) for individual Sm25 and Sm29 RDTs as well as when positivity by either RDT was considered. Sensitivity calculations were conducted based on 50 *S. mansoni* egg-positive sera as well as the contrived whole blood and DBS generated from the same set of 50 sera. Specificity calculations were conducted based on 139 sera (50 normal human sera, 50 *S. haematobium* egg-positive sera, and 39 parasite cross-reactor sera) and the contrived whole blood and DBS generated from the same set of 139 sera. **(A)** Values highlighted in yellow represent sensitivity between 10 and 25 percentage points below the TPP goal of 0.95 and values highlighted in red represent sensitivity lower than 25 percentage points below 0.95. **(B)** Values highlighted in dark green represent specificity that meets the TPP goal of 0.80. **(C, D)** Matrix equivalency showing the Lumos value (relative units) for 50 *S. mansoni* egg-positive samples when serum, whole blood, or DBS was the matrix for Sm25 RDTs **(C)** and Sm29 RDTs **(D)**. Individual points represent sample measurements. Bars indicate the group mean, and error bars represent the mean +/- 1 standard deviation. The dashed red line indicates the 0.01 RU positivity threshold. Values are displayed on a pseudo-logarithmic y-axis, which behaves approximately linearly around zero and increasingly logarithmically at larger values. This transformation improves visualization of values clustered near zero while retaining zero and slightly negative observations. **(E)** Venn diagram illustrating which of the 50 egg-positive *S. mansoni* samples had Lumos values above the positivity cut-off when serum was the matrix for the Sm25 RDT, Sm29 RDT, both, or neither.

Based on Lumos data, RDT sensitivity using serum as the matrix was higher than with either whole blood or DBS. Across all three matrices, Sm29 RDTs were consistently more sensitive than Sm25 RDTs alone. However, sensitivity increased when considering reactivity on either RDT as a positive test. The highest observed sensitivity based on Lumos data was 0.74 for positivity by either RDT using serum as the matrix (**Figure 2A**). While there were large differences in diagnostic sensitivity between the various RDTs and matrices, there were only slight differences in specificity using Lumos data. Specificity remained consistent, ranging from 0.94 to 0.99 for the various RDTs and matrices, even when positivity by either RDT was considered (**Figure 2B**).

We next analyzed matrix equivalency by comparing the Lumos values for the *S. mansoni* egg-positive sera. For Sm25, Lumos values differed significantly among matrices (χ^2^(2) = 20.3, p < 0.001). Descriptively, mean values were highest in serum, followed by DBS, and lowest in whole blood. Pairwise non-parametric comparisons showed significant differences between DBS and whole blood (adjusted p = 0.004), DBS and serum (adjusted p = 0.003), and whole blood and serum (adjusted p < 0.001).

For Sm29, Lumos values also differed significantly among matrices (χ^2^(2) = 20.8, p < 0.001). Descriptively, mean values were highest in serum, followed by DBS and whole blood. Pairwise non-parametric comparisons showed that serum differed significantly from both DBS and whole blood (adjusted p < 0.001 for both comparisons), while DBS and whole blood were not significantly different from each other. Across all matrices, Sm29 consistently showed higher mean Lumos values compared to Sm25 (**Figures 2C, D**). However, we found no significant correlation between egg count and Lumos value for both Sm25 and Sm29 RDTs (non-parametric, two-tailed Spearman p-values > 0.05; data not shown).

Furthermore, we visualized the overlap of which *S. mansoni* egg-positive sera were identified as positive by one RDT, both, or neither according to Lumos results. Only 17 (34%) sera were positive by both RDTs and there were nearly two times more samples picked up only by Sm29 (n=13) compared with Sm25 (n=7). Moreover, 13 (26%) of the sera from egg-positive persons were negative on both RDTs (**Figure 2E**).

### 3.2 Diagnostic sensitivity and specificity of the Sm25 and Sm29 RDTs by visual scoring

While the Lumos provides valuable quantification of test line intensity for comparisons between the two RDTs and three matrices, TPP requirements stipulate that tests should be able to operate equipment-free at the point of care. Consequently, we also included visual evaluation by two independent readers. Across all matrices and RDTs, restricting positivity criteria to only visual positives resulted in decreased sensitivity compared to Lumos. Conversely, including borderline positives resulted in increased sensitivity compared to Lumos (**Table 1**). Overall, trends mirrored the observations by Lumos, where Sm29 was more sensitive than Sm25 alone but considering positive results by either RDT increased sensitivity further. Of note, sensitivity by DBS when borderline positives were included was consistently much higher than all other measures, reaching 0.98 when positivity by either RDT was considered (**Table 1**). As readers were scoring cassettes when DBS was the matrix, they observed many instances of what appeared as a shadow near the test line that was difficult to distinguish from a faint line. This shadow was commonly counted as borderline positive during visual scoring but was often not positive by the Lumos reader.

**Table 1:** Sensitivity of Sm25 and Sm29 RDTs when evaluating different visual scoring methods compared to the Lumos reader.

TPP Goal >0.95							
		Serum		Whole Blood		DBS	
		Sensitivity	95% CI	Sensitivity	95% CI	Sensitivity	95% CI
Sm25	Lumos	0.48	0.35 - 0.62	0.24	0.14 - 0.37	0.42	0.29 - 0.56
	Visual - Positive Only	0.34	0.22 - 0.48	0.18	0.10 - 0.31	0.34	0.22 - 0.48
	Visual - Borderline Included	0.58	0.44 - 0.71	0.32	0.21 - 0.46	0.86	0.74 - 0.93
Sm29	Lumos	0.60	0.46 - 0.72	0.54	0.40 - 0.67	0.54	0.40 - 0.67
	Visual - Positive Only	0.58	0.44 - 0.71	0.44	0.31 - 0.58	0.48	0.35 - 0.62
	Visual - Borderline Included	0.66	0.52 - 0.78	0.60	0.46 - 0.72	0.86	0.74 - 0.93
Either	Lumos	0.74	0.60 - 0.84	0.64	0.50 - 0.76	0.64	0.50 - 0.76
	Visual - Positive Only	0.70	0.56 - 0.81	0.52	0.39 - 0.65	0.58	0.44 - 0.71
	Visual - Borderline Included	0.80	0.67 - 0.89	0.70	0.56 - 0.81	0.98	0.90 - 1.00

Diagnostic sensitivity was calculated for serum, whole blood, and DBS for individual Sm25 and Sm29 RDTs as well as when positivity by either RDT was considered. Positivity as determined by the Lumos reader cutoff is also included for reference. Sensitivity calculations were conducted based on 50 *S. mansoni* egg-positive sera as well as the contrived whole blood and DBS generated from the same set of 50 sera. For each value, the 95% confidence interval (CI) is shown. A value highlighted in dark green represents sensitivity that meets the TPP goal of 0.95, a value highlighted in light green represents sensitivity within 10 percentage points below the TPP goal of 0.95, a value highlighted in yellow represents sensitivity between 10 and 25 percentage points below 0.95, and a value highlighted in red represents sensitivity lower than 25 percentage points below 0.95.

For visual specificity, values calculated based on Visual – Positive Only remained high: ranging from 0.98 to 0.99 for all matrices and RDTs, including when positivity by either RDT was considered (**Table 2**). When borderline positives were included, specificity was highest for Sm25, followed by Sm29 then positivity by either RDT. Across matrices, specificity was overall highest for whole blood, followed by serum, with the largest decreases noted for DBS. The lowest observed specificity was 0.76 when borderline positives were included for DBS and positivity by either RDT was considered (**Table 2**).

**Table 2:** Specificity of Sm25 and Sm29 RDTs when evaluating different visual scoring methods compared to the Lumos reader.

TPP Goal >0.80							
		Serum		Whole Blood		DBS	
		Specificity	95% CI	Specificity	95% CI	Specificity	95% CI
Sm25	Lumos	0.99	0.95 - 1.00	0.99	0.95 - 1.00	0.99	0.96 - 1.00
	Visual - Positive Only	0.99	0.96 - 1.00	0.99	0.96 - 1.00	0.98	0.94 - 1.00
	Visual - Borderline Included	0.95	0.90 - 0.98	0.99	0.96 - 1.00	0.82	0.74 - 0.93
Sm29	Lumos	0.95	0.90 - 0.98	0.97	0.93 - 0.99	0.96	0.91 - 0.98
	Visual - Positive Only	0.99	0.95 - 1.00	0.99	0.95 - 0.99	0.99	0.96 - 1.00
	Visual - Borderline Included	0.84	0.77 - 0.89	0.86	0.79 - 0.91	0.81	0.74 - 0.87
Either	Lumos	0.94	0.89 - 0.97	0.96	0.92 - 0.99	0.96	0.91 - 0.98
	Visual - Positive Only	0.99	0.95 - 1.00	0.99	0.95 - 1.00	0.98	0.94 - 0.99
	Visual - Borderline Included	0.81	0.74 - 0.87	0.86	0.79 - 0.91	0.76	0.68 - 0.82

Diagnostic specificity was calculated for serum, whole blood, and DBS for individual Sm25 and Sm29 RDTs as well as when positivity by either RDT was considered. Positivity as determined by the Lumos reader cutoff is also included for reference. Specificity calculations were conducted based on 139 sera (50 normal human sera, 50 *S. haematobium* egg-positive sera, and 39 parasite cross-reactor sera) and the contrived whole blood and DBS generated from the same set of 139 sera. For each value, the 95% confidence interval (CI) is shown. A value highlighted in dark green represents specificity that meets the TPP goal of 0.80 while values highlighted in light green represent specificities within 10 percentage points below 0.80.

We focused specifically on whole blood evaluated by visual readers as this would be the most applicable to tests run at the point-of-care. We generated 2x2 contingency tables and calculated the overall diagnostic accuracy, positive predictive value (PPV), and negative predictive value (NPV) for both RDTs compared to Kato-Katz results (**Tables 3**–**5**). For these calculations, we determined positivity as a test where at least one reader counted the result as borderline positive because this metric resulted in the highest sensitivity while still allowing specificity to meet TPP criteria. Based on this dataset, the Sm25 RDTs had a much higher PPV compared to Sm29, while the Sm29 RDTs had a slightly higher NPV compared to Sm25. Overall, the diagnostic accuracy of Sm25 was slightly higher than Sm29 (**Tables 3**–**5**).

**Table 3:** 2x2 contingency table comparing the Sm25 RDT to the Kato-Katz method.

		Sm25 RDT	
		Negative	Positive
Kato-Katz	Negative	138	1
	Positive	34	16

50 *S. mansoni* samples were egg-positive by Kato-Katz. 139 samples were considered negative by Kato-Katz (50 normal human sera (NHS), 50 *S. haematobium* egg-positive sera, and 39 parasite cross-reactor sera) but these samples did not have matched Kato-Katz results. All NHS, *S. haematobium* and 20 parasite cross-reactor samples were from persons living in *S. mansoni non*-endemic areas, but the 19 samples from individuals positive via clinical blood culture for *Leishmania donovani* lack complete travel/living histories. Positivity by the Sm25 RDT was based on contrived whole blood results scored by two visual readers when at least one reader scored the test as borderline positive.

**Table 4:** 2x2 contingency table comparing the Sm29 RDT to the Kato-Katz method.

		Sm29 RDT	
		Negative	Positive
Kato-Katz	Negative	119	20
	Positive	20	30

50 *S. mansoni* samples were egg-positive by Kato-Katz. 139 samples were considered negative by Kato-Katz (50 normal human sera (NHS), 50 *S. haematobium* egg-positive sera, and 39 parasite cross-reactor sera) but these samples did not have matched Kato-Katz results. All NHS, *S. haematobium* and 20 parasite cross-reactor samples were from persons living in *S. mansoni non*-endemic areas, but the 19 samples from individuals positive via clinical blood culture for *Leishmania donovani* lack complete travel/living histories. Positivity by the Sm25 RDT was based on contrived whole blood results scored by two visual readers when at least one reader scored the test as borderline positive.

**Table 5:** Diagnostic accuracy, positive predictive value, and negative predictive value of the Sm25 and Sm29 RDTs when compared to Kato-Katz results based on this dataset. 50 *S. mansoni* samples were egg-positive by Kato-Katz.

Metric	Sm25 RDT	Sm29 RDT
Diagnostic Accuracy	0.82	0.79
Positive Predictive Value	0.94	0.60
Negative Predictive Value	0.80	0.86

139 samples were considered negative by Kato-Katz (50 normal human sera (NHS), 50 *S. haematobium* egg-positive sera, and 39 parasite cross-reactor sera) but these samples did not have matched Kato-Katz results. All NHS, *S. haematobium* and 20 parasite cross-reactor samples were from persons living in *S. mansoni non*-endemic areas, but the 19 samples from individuals positive via clinical blood culture for *Leishmania donovani* lack complete travel/living histories. Positivity by each RDT was based on contrived whole blood results scored by two visual readers when at least one reader scored the test as borderline positive.

### 3.3 Comparison of the Sm25 and Sm29 RDTs to recombinant antigen ELISAs

To investigate whether the low sensitivity of the Sm25 and Sm29 RDTs was related to the RDT test platform, we evaluated the same set of sera on ELISAs that test for IgG1 antibodies against Sm25 and Sm29. We also used an ELISA that tests for IgG1 antibodies against TSP2, a recombinant antigen from an *S. mansoni* tetraspanin protein, to see if this additional recombinant antigen might detect infections missed by Sm25 and Sm29 (Tran et al., 2006) (Wang et al., in preparation).

**Figure 3:**
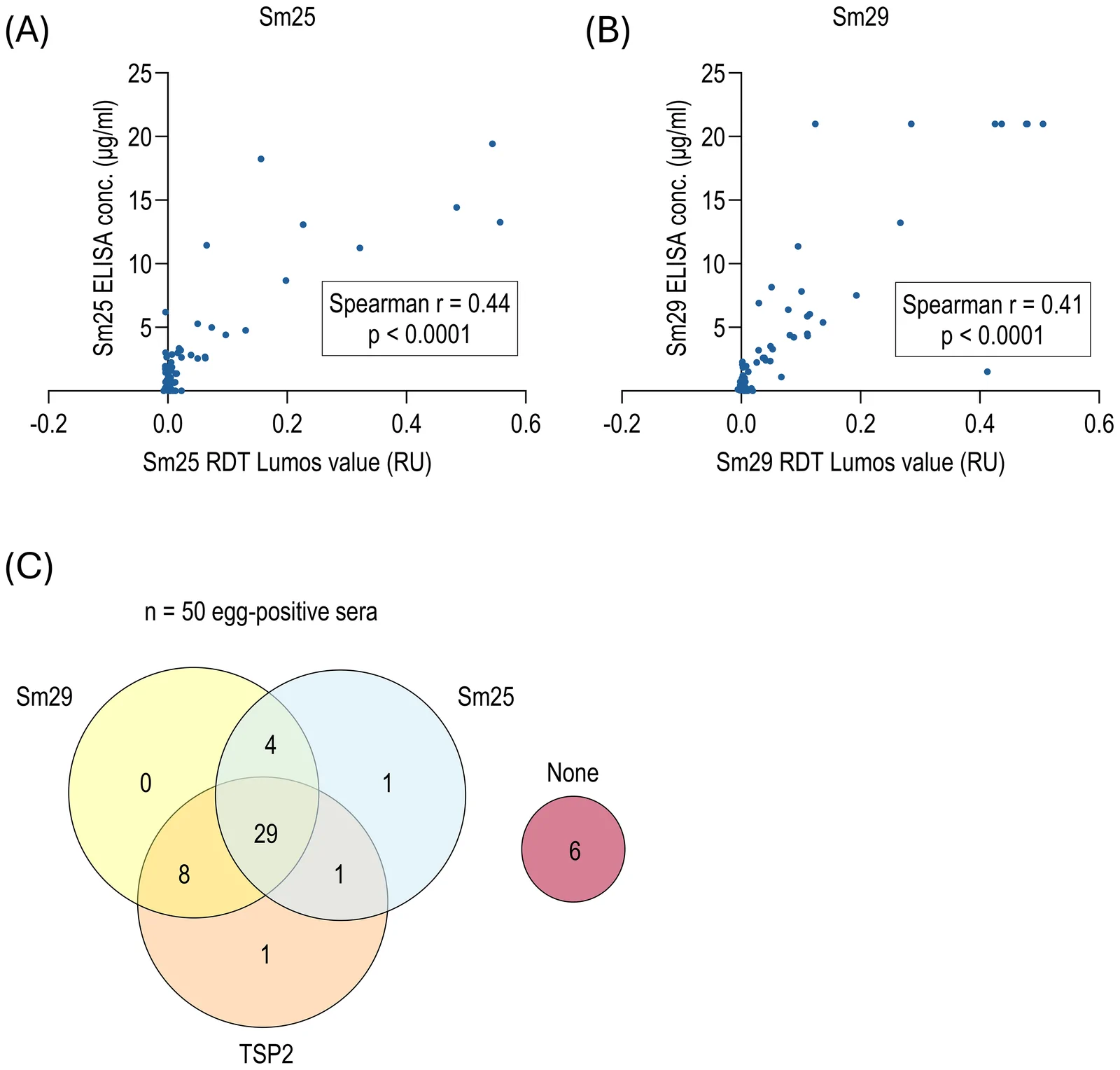
Comparison of the Sm25 and Sm29 RDTs to Sm25, Sm29 and TSP2 ELISAs. Comparison of RDT Lumos values (relative units) to corresponding ELISA concentration (μg/ml) for Sm25 **(A)** and Sm29 **(B)** based on 189 unique sera (50 *S. mansoni* egg-positive sera, 50 normal human sera, 50 *S. haematobium* egg-positive sera, and 39 parasite cross-reactor sera). Spearman r and p-values from a non-parametric, two-tailed Spearman correlation analysis are shown. **(C)**. Venn diagram illustrating which of the 50 egg-positive *S. mansoni* samples were positive by none, one, two, or all three of the Sm25, Sm29, and TSP2 recombinant antigen ELISAs.

To compare performance of the Sm25 or Sm29 RDT to the ELISA of the corresponding antigen, we plotted the ELISA concentration against the RDT Lumos value for each sample. A non-parametric, two-tailed Spearman correlation analysis of these data showed r values of 0.44 (p < 0.0001) and 0.41 (p < 0.0001) for Sm25 and Sm29, respectively (**Figures 3A, B**), indicating a statistically significant relationship between Lumos value and ELISA reactivity.

We also conducted a concordance analysis in which concordance was defined as both the ELISA and the RDT for a specific antigen having the same result for the same sample, whether both positive or both negative. Concordance between the Sm25 RDT and Sm25 ELISA was 86% (163/189) and concordance between the Sm29 RDT and Sm29 ELISA was 87% (165/189) (data not shown).

Additionally, we generated a Venn diagram to visualize how many *S. mansoni* egg-positive samples were positive by none, one, two, or all three ELISAs. From this analysis, 29 (58%) of the sera were positive by all three ELISAs. If TSP2 is removed from analysis, 33 (66%) of samples were identified as positive by both Sm25 and Sm29 ELISAs (**Figure 3C**), which is nearly three times more overlap than with the RDTs (26%) (**Figure 2E**). Despite this increase in overlap, six (12%) sera were still not positive by any of the three recombinant antigen ELISAs (**Figure 3C**).

### 3.4 MBA analysis of the six *S. mansoni* egg-positive sera that were not detected via ELISA

**Figure 4:**
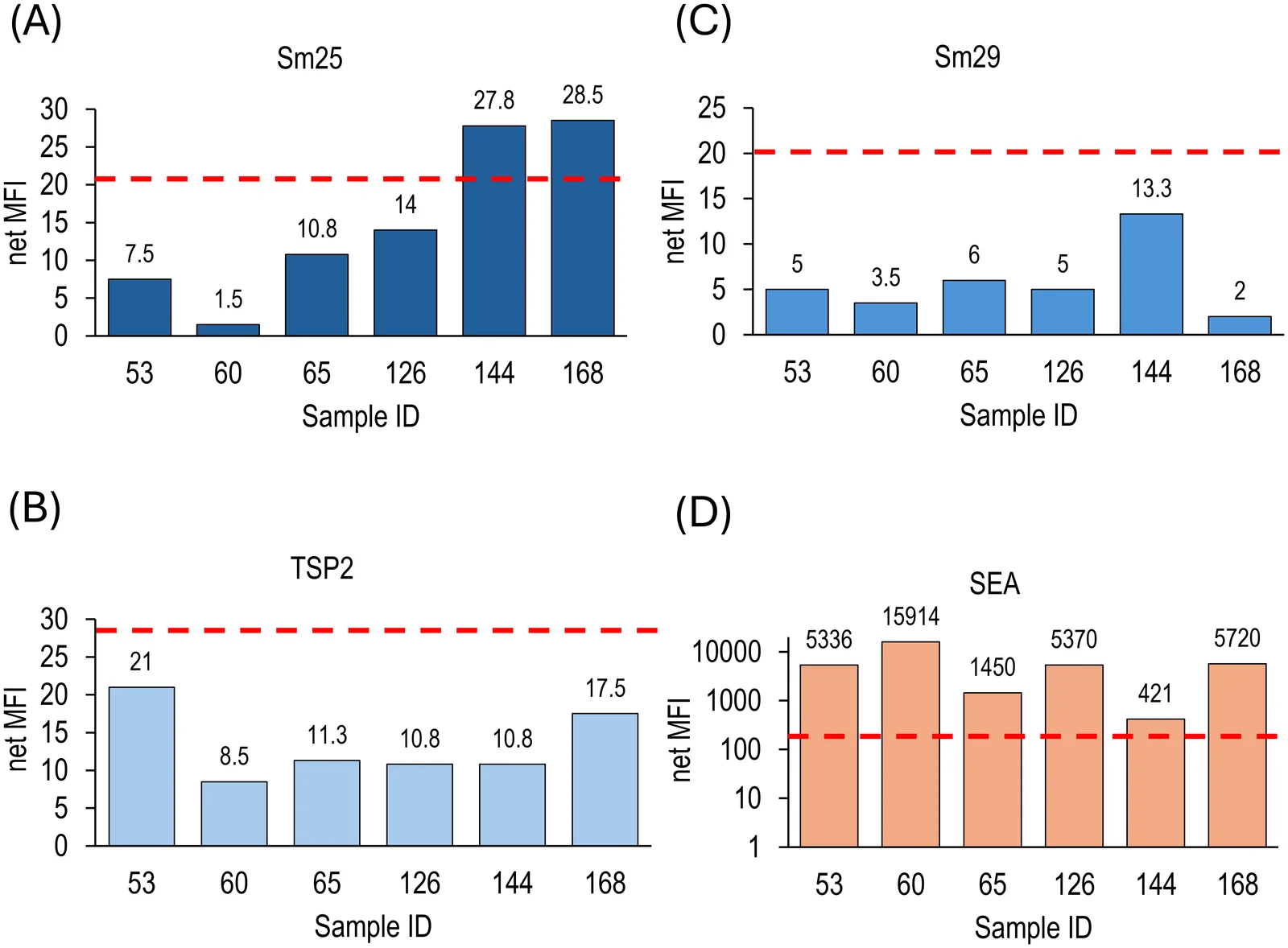
MBA evaluation of the six sera that were negative by all three recombinant antigen ELISAs. Sera were evaluated for antibodies against Sm25 **(A)**, Sm29 **(B)**, TSP2 **(C)**, and SEA **(D)** using MBA. Samples, as listed by their blinded study ID, were run in duplicate and the net mean fluorescent intensity (MFI) is displayed above each bar. The positivity cut-off for each specific antigen is indicated using a red dotted line.

As six *S. mansoni* egg-positive sera were still not detected by any of the recombinant antigen ELISAs, we also evaluated them using an additional method: the MBA. Using MBA, we detected IgG and IgG4 antibody responses to the Sm25, Sm29, and TSP2 recombinant antigens, as well as *S. mansoni* SEA. Using MBA, these six egg-positive sera were also negative for all three recombinant antigens, aside from two sera that were just over the positivity cutoff for Sm25 (**Figures 4A–C**). However, when we evaluated the six sera using SEA, we found that all were positive (**Figure 4D**). Data across the RDTs (both visual and by Lumos), ELISAs, and MBA, as well as the average EPG in stool for these six sera are summarized in **Table 6**. Of note, three sera were borderline positive on RDT by one or both operators and one was positive by Lumos for the Sm25 RDT (**Table 6**).

**Table 6:** Summary of serum data for the six *S. mansoni* egg-positive samples that tested negative by all three ELISAs.

		Sm25 RDT results			Sm29 RDT results			ELISA results			MBA results			
Sample ID	Avg. EPG	Visual operator 1	Visual operator 2	Lumos	Visual operator 1	Visual operator 2	Lumos	Sm25	Sm29	CD63	Sm25	Sm29	CD63	SEA
53	169	0	4	0	0	0	0	0	0	0	0	0	0	1
60	32	0	0	0	0	0	0	0	0	0	0	0	0	1
65	92	0	4	0	4	0	0	0	0	0	0	0	0	1
126	40	0	0	0	0	0	0	0	0	0	0	0	0	1
144	44	0	0	0	0	0	0	0	0	0	1	0	0	1
168	388	4	4	1	0	0	0	0	0	0	1	0	0	1

Visual data from both operators and Lumos data from Sm25 and Sm29 RDTs, ELISA results, and MBA results for these samples are summarized such that 0=negative, 1=positive (green), and 0.5=borderline positive (yellow; visual RDT results only). In the average eggs per gram (EPG) column, the average number of *S. mansoni* eggs from two different Kato-Katz slides across three separate stools are shown for each individual. Egg counts of 1–99 indicate a light infection intensity and egg counts of 100–399 indicate a moderate intensity infection.

### 3.5 Stability of the Sm25 and Sm29 RDT test line signal

**Figure 5:**
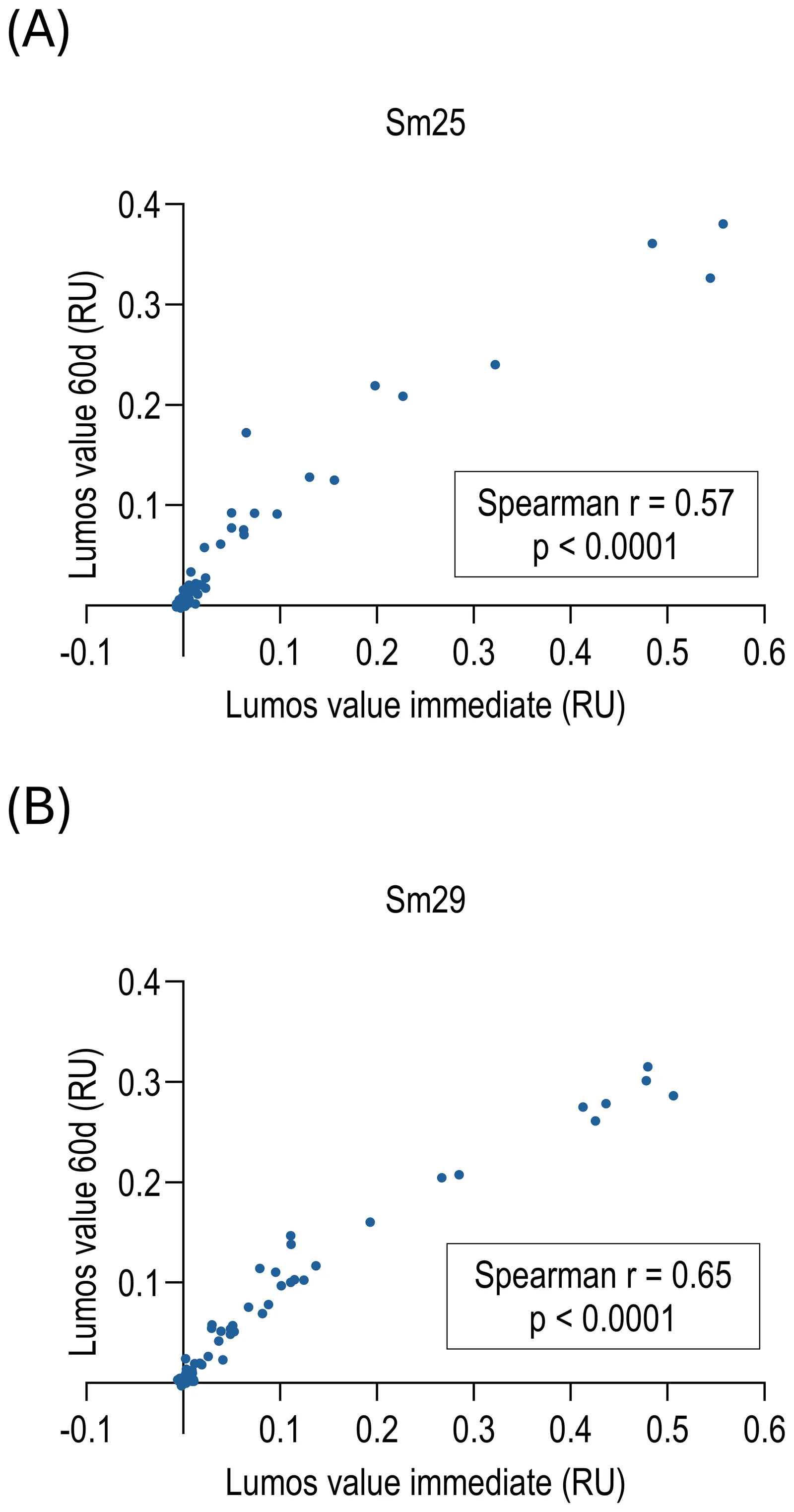
Stability of the test line signal on Sm25 and Sm29 RDTs. Test line signal on Sm25 **(A)** and Sm29 **(B)** cassettes run with 189 unique sera (50 *S. mansoni* egg-positive sera, 50 normal human sera, 50 *S. haematobium* egg-positive sera, and 39 parasite cross-reactor sera) was measured using the Lumos reader immediately and again after approximately 60 days. Spearman r and p-values from non-parametric, two-tailed Spearman correlation analyses are shown.

Finally, we evaluated the test line stability on Sm25 and Sm29 RDT cassettes by plotting the Lumos value after 60 days of storage at constant temperature (~25 °C) against the initial Lumos value. A non-parametric, two-tailed Spearman correlation analysis of these data was conducted. Spearman r values of 0.57 (p < 0.0001) and 0.65 (p < 0.0001) were obtained for Sm25 and Sm29, respectively (**Figures 5A,B**), indicating a statistically significant correlation between the initial Lumos values and the values after 60 days. To investigate qualitative result concordance, we defined concordance as the same cassette being positive or negative at both timepoints. We found a 95% and 97% concordance for Sm25 and Sm29, respectively (data not shown).

## 4 Discussion

To investigate whether Sm25 and Sm29 RDT prototypes met TPP criteria for an initial screening tool, we evaluated the tests using three different matrices: serum, whole blood and DBS. The specificity of individual RDTs surpassed TPP criteria across all matrices. It was particularly notable that these tests were highly specific with respect to *S. haematobium*, another schistosome species that causes urogenital schistosomiasis. While cross-reactivity against a handful of other parasites was minimal, future work could evaluate additional parasites, including soil-transmitted helminths and *Plasmodium* spp. These infections are often co-endemic with schistosomiasis and could impact specificity (Perera et al., 2024), but were not evaluated as part of this study due to specimen availability.

Using both a Lumos automated reader and two visual readers, we found that Sm29 RDTs were more sensitive than Sm25 RDTs across all three matrices. While considering positivity by either RDT increased sensitivity and allowed for identification of more infections than individual RDTs, sensitivity still fell short of TPP goal regardless of matrix. The only instance when sensitivity met TPP goals was when visual borderline results were counted as positive by either RDT using DBS as the matrix. This increase was likely a result of the “shadows” observed with the DBS matrix. While this phenomenon warrants follow-up investigation, we hypothesize that may have been due to the lysis of RBCs during elution in the proprietary chase buffer that contains detergent.

While Lumos provides valuable quantitative data, visual readings would be conducted at the POC, consistent with TPP criteria that specify tests should be equipment-free (WHO, 2021). Across all matrices and both RDTs, only counting visual positives decreased sensitivity compared to Lumos, while including borderline positives increased sensitivity. These differences suggest that the visual scoring process could benefit from standardization to overcome the subjectivity associated with scoring a line as positive vs. borderline, which has previously created controversy in other *S. mansoni* tests (Peralta and Cavalcanti, 2018). Though counting borderline results as positive did cause trade-offs in terms of reduced specificity, specificity still met TPP criteria for all analyses except when borderline results from positivity by either RDT using DBS were counted.

When comparing matrices, we found sensitivity was highest with serum. Notably, when the matrix was whole blood, Sm25 RDTs had a large decrease in sensitivity. This is consistent with the observation that this matrix/antigen combination had the lowest average Lumos signal of any combination we evaluated. Across matrices, Sm29 Lumos signal was consistently higher compared to Sm25. Overall, matrix equivalency comparisons found that serum had the highest mean Lumos values, followed by DBS then whole blood. As fingerstick whole blood would be the desired matrix for use of these RDTs in the field, increasing the test line intensity for whole blood specimens will be an important consideration in the development of future RDT prototypes.

As whole blood evaluated visually would be the most applicable to TPP criteria, we focused on this combination particularly. When visual borderline results were counted as positive by either RDT using whole blood as the matrix, sensitivity only reached 0.70: far short of the 0.95 TPP goal. Looking at each RDT individually, we found that Sm25 had the highest PPV and overall diagnostic accuracy, while Sm29 had the highest NPV for this dataset. As predictive values are not intrinsic to the test and depend on the population, a field evaluation in areas of low prevalence will be key components in the evaluation of more promising RDT prototypes to ensure that they are useful in low-prevalence areas.

The use of contrived whole blood and contrived DBS was necessary to allow for paired comparisons between matrices from a single individual without conducting a large-scale field study. However, use of contrived specimens has limitations and may result in an overestimate of performance when compared to whole blood or DBS specimens collected and tested in a field setting. Though contrived blood has been used to evaluate other antibody-detection lateral flow assays (Gwyn et al., 2016), it lacks components such as clotting factors which may have affected its flow through the cassette and contributed to the low sensitivity we observed with this matrix (Mei et al., 2001). Consequently, if future RDT prototypes exhibit promising results based on contrived specimens in a laboratory setting, additional testing should be conducted using blood samples and DBS taken at the point-of-care, in comparison to blood and sera collected from the same individual.

Despite the increased ability of the ELISAs to detect egg-positive persons compared to the RDTs, the recombinant antigens were still not able to identify all *S. mansoni* egg-positives. Further, the egg-positive samples missed by ELISA largely remained negative by recombinant antigens on MBA. However, the MBA found that all six samples were positive for antibodies against SEA, which is a complex mix of native antigens. This confirms that while these egg-positive individuals lacked detectable antibody responses to the recombinant antigens, they had mounted an anti-schistosome humoral immune response. Though SEA is useful in this context, production, which involves maintaining the schistosome life cycle in snails and rodents, cannot keep up with global demand and is prone to batch-to-batch differences. By contrast, recombinant antigens can be commercially produced in a cost-effective manner. Together, these data emphasize a case for additional recombinant antigen discovery to capture the infections we are missing using current recombinant antigens, regardless of test platform. Our study was limited in that all the *S. mansoni* egg-positive specimens we evaluated were from western Kenya; inclusion of specimens from other endemic regions could reveal gaps in detection that we could not identify here. Evaluation of more geographically diverse panels using these recombinant antigens will be a key future direction to determine whether this observation is consistent in other populations.

Notably, these RDT prototypes required a second buffer addition step. While management of multiple buffer additions was possible in a controlled laboratory setting with multiple operators, conditions in the field may prevent this from being done effectively. DDTD has explored test method modifications for other RDTs that remove this second buffer step, which would effectively reduce the complexity of running these RDTs in a field setting.

Lastly, the two-month RDT result stability we observed far exceeds the TPP stability goal of 0.5 hours (WHO, 2021). A highly stable test line would allow for cassettes to be brought to a central location and read by supervisory laboratory staff or shipped to partners for quality control. Furthermore, this would allow the tests to operate equipment-free in the field to meet TPP requirements while allowing for later quantitative analysis using a cassette reader like the Lumos. While some test strips had molded after 60 days, these were isolated incidents and may not have been an issue if the cassettes had dried completely before storage.

Overall, these novel Sm25 and Sm29 RDTs represent the first prototypes toward a rapid antibody detection test that could serve as an initial screening tool for the *S. mansoni* transmission elimination and surveillance use case. While these tests meet TPP specificity and stability criteria, their sensitivity falls short of TPP goals. If sensitivity can be increased, future RDT prototypes could also act as a quick, low-technology tool for mapping areas that lack historical data on schistosomiasis prevalence, expanding the potential use cases. Our evaluation of the effect of sample matrix on RDT performance will provide a framework for development of a new generation of RDTs with increased sensitivity.

## Data Availability

The raw data supporting the conclusions of this article will be made available by the authors, without undue reservation.
